# Quantitative Muscle MRI in Patients with Neuromuscular Diseases—Association of Muscle Proton Density Fat Fraction with Semi-Quantitative Grading of Fatty Infiltration and Muscle Strength at the Thigh Region

**DOI:** 10.3390/diagnostics11061056

**Published:** 2021-06-08

**Authors:** Sarah Schlaeger, Nico Sollmann, Agnes Zoffl, Edoardo Aitala Becherucci, Dominik Weidlich, Elisabeth Kottmaier, Isabelle Riederer, Tobias Greve, Federica Montagnese, Marcus Deschauer, Benedikt Schoser, Claus Zimmer, Dimitrios C. Karampinos, Jan S. Kirschke, Thomas Baum

**Affiliations:** 1 Department of Diagnostic and Interventional Neuroradiology, School of Medicine, Klinikum rechts der Isar, Technical University of Munich, Ismaninger Str. 22, 81675 Munich, Germany; agnes.zoffl@gmail.com (A.Z.); edoardobecherucci@gmail.com (E.A.B.); elisabeth.klupp@tum.de (E.K.); isabelle.rieder@tum.de (I.R.); claus.zimmer@tum.de (C.Z.); jan.kirschke@tum.de (J.S.K.); thomas.baum@tum.de (T.B.); 2 TUM-Neuroimaging Center, Klinikum rechts der Isar, Technical University of Munich, 81675 Munich, Germany; 3 Department of Diagnostic and Interventional Radiology, University Hospital Ulm, Albert-Einstein-Allee 23, 89081 Ulm, Germany; 4 Department of Diagnostic and Interventional Radiology, School of Medicine, Klinikum rechts der Isar, Technical University of Munich, Ismaninger Str. 22, 81675 Munich, Germany; dominik.weidlich@tum.de (D.W.); dimitrios.karampinos@tum.de (D.C.K.); 5 Department of Neurosurgery, University Hospital, LMU Munich, Marchioninistr. 15, 81377 Munich, Germany; tobias.greve@med.uni-muenchen.de; 6 Department of Neurology, Friedrich-Baur-Institute, LMU Munich, Ziemssenstr. 1a, 80336 Munich, Germany; federica.montagnese@med.uni-muenchen.de (F.M.); benedikt.schoser@med.uni-muenchen.de (B.S.); 7 Department of Neurology, School of Medicine, Klinikum rechts der Isar, Technical University of Munich, Ismaninger Str. 22, 81675 Munich, Germany; marcus.deschauer@mri.tum.de

**Keywords:** chemical shift encoding-based water-fat MRI, neuromuscular diseases, proton density fat fraction, quantitative MRI, thigh musculature, muscle strength

## Abstract

(1) Background and Purpose: The skeletal muscles of patients suffering from neuromuscular diseases (NMD) are affected by atrophy, hypertrophy, fatty infiltration, and edematous changes. Magnetic resonance imaging (MRI) is an important tool for diagnosis and monitoring. Concerning fatty infiltration, T_1_-weighted or T_2_-weighted DIXON turbo spin echo (TSE) sequences enable a qualitative assessment of muscle involvement. To achieve higher comparability, semi-quantitative grading scales, such as the four-point Mercuri scale, are commonly applied. However, the evaluation remains investigator-dependent. Therefore, effort is being invested to develop quantitative MRI techniques for determination of imaging markers such as the proton density fat fraction (PDFF). The present work aims to assess the diagnostic value of PDFF in correlation to Mercuri grading and clinically determined muscle strength in patients with myotonic dystrophy type 2 (DM2), limb girdle muscular dystrophy type 2A (LGMD2A), and adult Pompe disease. (2) Methods: T_2_-weighted two-dimensional (2D) DIXON TSE and chemical shift encoding-based water-fat MRI were acquired in 13 patients (DM2: *n* = 5; LGMD2A: *n* = 5; Pompe disease: *n* = 3). Nine different thigh muscles were rated in all patients according to the Mercuri grading and segmented to extract PDFF values. Muscle strength was assessed according to the British Medical Research Council (BMRC) scale. For correlation analyses between Mercuri grading, muscle strength, and PDFF, the Spearman correlation coefficient (r_s_) was computed. (3) Results: Mean PDFF values ranged from 7% to 37% in adults with Pompe disease and DM2 and up to 79% in LGMD2A patients. In all three groups, a strong correlation of the Mercuri grading and PDFF values was observed for almost all muscles (*r_s_* > 0.70, *p* < 0.05). PDFF values correlated significantly to muscle strength for muscle groups responsible for knee flexion (*r_s_* = −0.80, *p* < 0.01). (4) Conclusion: In the small, investigated patient cohort, PDFF offers similar diagnostic precision as the clinically established Mercuri grading. Based on these preliminary data, PDFF could be further considered as an MRI-based biomarker in the assessment of fatty infiltration of muscle tissue in NMD. Further studies with larger patient cohorts are needed to advance PDFF as an MRI-based biomarker in NMD, with advantages such as its greater dynamic range, enabling the assessment of subtler changes, the amplified objectivity, and the potential of direct correlation to muscle function for selected muscles.

## 1. Introduction

Neuromuscular diseases (NMD) represent a clinically very heterogeneous group of inherited or acquired disorders with alteration of the muscle tissue itself or of their innervating nerves [1,2,3]. Recent progress in genetic diagnostics provides new insights into the underlying pathogenesis [4,5]. Developments in gene therapy and pharmacogenetics start improving treatment and modifying disease courses [4,5].

The musculature of patients suffering from NMD is mainly affected by atrophy, hypertrophy, fatty infiltration, and edematous changes. Thus, magnetic resonance imaging (MRI), providing a high soft-tissue contrast, has evolved as an important tool for diagnosis and monitoring over the past decades [6,7,8,9]. Specifically, MRI allows the spatially resolved detection of characteristic patterns of muscle involvement [10,11,12,13,14,15,16,17,18,19,20,21], helps to limit the range of differential diagnoses, and guides muscle biopsies as well as genetic analysis. Additionally, MRI facilitates monitoring of disease progression and the response to modern treatment [5,22,23,24]. Routinely acquired MRI, including T_1_-weighted or fat-suppressed T_2_-weighted sequences, enables qualitative assessments of the patients’ muscle involvement [23,25,26,27,28,29]. Recently, T_2_-weighted two-dimensional (2D) DIXON turbo spin echo (TSE) imaging has been proposed for a simultaneous qualitative assessment of fatty infiltration and edematous alterations [30,31,32,33].

By applying semi-quantitative grading scales on qualitative imaging sequences, such as the Mercuri scale for fatty infiltration [24] or the Morrow scale for edematous changes [34], assessment of alterations in muscle tissue becomes more comparable and standardized among different examinations, readers, or patient groups. However, the semi-quantitative grading remains dependent on the reader’s judgment and experience and stays rather subjective because of non-harmonized scoring and reporting techniques [23]. Therefore, the evaluation of qualitative imaging data lacks reproducibility and reliability, which impacts its true value in the clinical setting. Subtle muscle tissue alterations, especially present at early disease stages, are not precisely assessable by qualitative imaging data.

To overcome these gaps of the current MRI assessments of muscle alterations in NMD and to foster more robust characterizations of subtle pathological changes, efforts have been made in the development of quantitative imaging techniques, particularly related to the proton density fat fraction (PDFF) and water T_2_ (T_2w_) determination as imaging markers for fatty infiltration and edematous alterations, respectively. Specifically, PDFF is defined as the ratio of density from mobile triglyceride protons and total density from mobile triglycerides and mobile water [35]. PDFF represents the degree of fatty infiltration in the muscle tissue [36]. Additionally, PDFF is seen as a marker for the physiological functionality of the muscle tissue as PDFF is inversely correlating with muscle strength [37,38]. Furthermore, PDFF has been shown to be rather insensitive to changes in acquisition parameters, different scanner vendors, and field strength inhomogeneities due to its fundamental tissue-property character [35]. PDFF mapping is based on chemical shift encoding-based water-fat MRI [35,36,39].

To date, there is still scarce data comparing qualitative and quantitative MRI regarding the diagnostic performance for assessment of fatty infiltration in association with clinically measured muscle strength, which is mostly due to the rare incidence of NMD. Most of the MRI data in NMD concentrates on dystrophinopathies, facioscapulohumeral dystrophy (FSHD), and myotonic dystrophy type 1 (DM1), with only few studies reporting data from multiple types of NMD simultaneously [23,40].

We hypothesized that PDFF offers comparable diagnostic precision as the clinically established Mercuri grading in patients suffering from three different types of NMD (myotonic dystrophy type 2 (DM2), limb girdle muscular dystrophy type 2A (LGMD2A), and adult Pompe disease). Thus, the purpose of the present work is to assess the correlation of the routinely performed, semi-quantitative grading of fatty infiltration and clinically determined muscle strength with PDFF.

## 2. Materials and Methods

### 2.1. Patients and Study Setup

Thirteen patients with NMD were included. Five patients suffered from DM2, five patients from LGMD2A, and three patients from adult Pompe disease (please refer to Section 3.1 for patient details). The diagnosis was based on muscle biopsies and genetic testing. All patients underwent detailed neurological examination, including assessment of muscle strength and MRI of the thigh region bilaterally during the same study visit.

### 2.2. Clinical Examination

The patients underwent detailed clinical examinations according to a standardized protocol by a neurologist, including testing of sensory and motor function, reflexes, mental status, coordination, and cranial nerve function. Muscle strength was assessed according to the British Medical Research Council (BMRC) scale, with a score of 5 defined as normal power and 0 defined as complete paralysis [41].

### 2.3. Magnetic Resonance Imaging

#### 2.3.1. Image Acquisition

The bilateral thigh regions were scanned on a 3-Tesla MRI scanner (Ingenia, Philips Healthcare, Best, The Netherlands), acquiring consecutive axial stacks to cover the whole thigh region from the hip down to the cranial edge of the patella on both sides. The built-in 12-channel posterior coil and a 16-channel anterior coil were used for scanning.

The scanning protocol included a T_2_-weighted 2D DIXON TSE sequence for qualitative assessment of the muscles [32] and chemical shift encoding-based water-fat MRI, employing a six-echo three-dimensional (3D) spoiled gradient echo sequence (Table 1). The sequence acquired the six echoes in a single repetition time (TR) using non-flyback (bipolar) read-out gradients. A flip angle of 3° was used to minimize T_1_ bias effects and B1 confounding effects [39,42]. Potential differences between coil elements concerning the B1 receive were normalized during sensitivity encoding (SENSE) reconstruction using the whole-body coil as a reference.

#### 2.3.2. Postprocessing

Water-fat separation of the six-echo 3D spoiled gradient echo sequence was performed online using the vendor’s mDIXON algorithm, which applies a complex-based water-fat decomposition using a single T_2_* correction and a pre-calibrated fat spectrum, accounting for the presence of the multiple peaks in the fat spectrum. B0 inhomogeneities were modelled during postprocessing [30]. The PDFF map was then computed as the ratio of the fat signal over the sum of fat and water signals [35].

### 2.4. Evaluation of Imaging Data

#### 2.4.1. Semi-Quantitative Assessment

The musculature of the thigh region was evaluated using the institutional Picture Archiving and Communication System (PACS) viewer (IDS7; Sectra AB, Linköping, Sweden) by a radiologist (I. R., seven years of experience) considering Mercuri grading scales [24,32,34]. The left and right thigh regions were assessed separately considering the following muscles: biceps femoris, gracilis, rectus femoris, sartorius, semimembranosus, semitendinosus, vastus intermedius, vastus lateralis, and vastus medialis muscles. One score was assigned per muscle and side. During evaluation, the radiologist was blinded to the distinct diagnosis of the patients and had no access to previous or follow-up image data, if any.

In detail, fatty infiltration was assessed via the four-point Mercuri grading scale with score 1 for muscles with normal appearance, score 2 for muscles with mild fatty infiltration (less than 30% of the volume of the original muscles), score 3 for muscles with moderate fatty infiltration (30 to 60% of the volume of the original muscles), and score 4 for muscles with severe fatty infiltration (greater than 60% of the volume of the original muscles) [24,32]. Grading was performed in axial slices of the T_2_-weighted 2D DIXON TSE sequence, with the fat image series being taken for grading of fatty infiltration. The entire field of view (FOV) was considered during evaluation, and in case of level-specific differences of scaling, the higher score was assigned for the respective muscle.

#### 2.4.2. Quantitative Assessment

**Figure 1:**
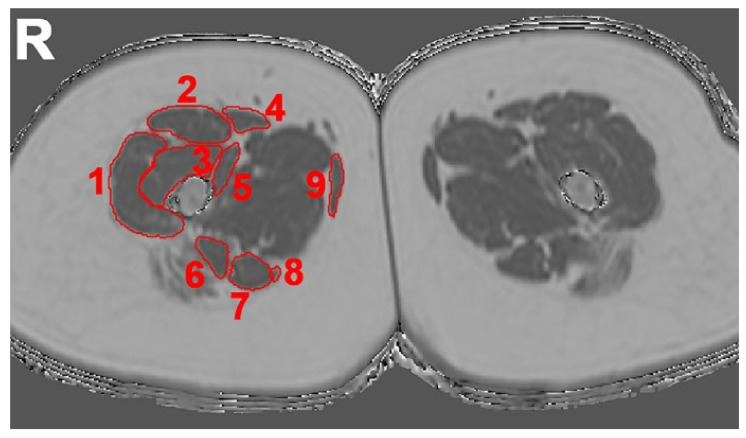
Segmentation masks on a representative axial slice of the PDFF map at the thigh region. Manual segmentation masks separately enclosing the vastus lateralis (**1**), rectus femoris (**2**), vastus intermedius (**3**), sartorius (**4**), vastus medialis (**5**), biceps femoris (**6**), semitendinosus (**7**), semimembranosus (**8**), and gracilis (**9**) muscles of the left and right thigh region, respectively. R (right).

Segmentations of the same thigh muscles that were used for semi-quantitative assessment were performed using the Medical Imaging Interaction Toolkit (http://mitk.org/wiki/The_Medical_Imaging_Interaction_Toolkit_(MITK), accessed on 15 April 2021; German Cancer Research Center, Division of Medical and Biological Informatics, Medical Imaging Interaction Toolkit, Heidelberg, Germany). Specifically, manual regions of interest (ROIs) were placed in axial slices of the PDFF maps derived from chemical shift encoding-based water-fat MRI to separately enclose the biceps femoris, gracilis, rectus femoris, sartorius, semimembranosus, semitendinosus, vastus intermedius, vastus lateralis, and vastus medialis muscles of the left and right thigh, respectively (Figure 1). Segmentation was performed on ten consecutive slices at the mid-thigh region, with the ROIs being placed at the outer muscle contour (Figure 1) by a radiologist (A. Z., three years of experience). Bilateral muscle-specific PDFF values were extracted from the ROIs.

### 2.5. Data Analysis and Statistics

Data analyses including statistics were performed with GraphPad Prism (version 6.0; GraphPad Software Inc., San Diego, CA, USA) and SPSS (version 20.0; IBM SPSS Statistics for Windows, IBM Corp., Armonk, NY, USA) (S.S., N.S. and T.B.). A *p*-value of 0.05 was set as the threshold for statistical significance.

Descriptive statistics were calculated for demographics, muscle strength, Mercuri scores derived from semi-quantitative assessment, and for PDFF values. The mean between both sides was calculated for BMRC scores of muscle strength. As PDFF values of the right and left side were not significantly different according to Wilcoxon signed-rank tests, PDFF values as well as scores of the Mercuri grading of both sides were averaged for further analysis. Obtained mean scores of the Mercuri grading and PDFF values were considered for each evaluated muscle separately. PDFF values were furthermore combined for muscles responsible for hip flexion (gracilis, rectus femoris, and sartorius muscles) and extension (biceps femoris, semimembranosus, and semitendinosus muscles) as well as knee flexion (biceps femoris, gracilis, sartorius, semimembranosus, and semitendinosus muscles) and extension (rectus femoris, vastus intermedius, vastus lateralis, and vastus medialis).

As all data were not normally distributed, age, scores of the Mercuri grading, and BMRC scores of muscle strength for hip and knee flexion and extension were compared between patients with DM2, LGMD2A, and Pompe disease using Kruskal–Wallis tests with Dunn’s multiple comparisons test for subsequent post-hoc testing. Moreover, muscle-specific PDFF values were analogously compared between patients with different NMD using Kruskal–Wallis tests with Dunn’s multiple comparisons test for post-hoc testing. *P*-values derived from Kruskal–Wallis tests are reported together with multiplicity adjusted *p*-values derived from Dunn’s tests (to be able to report *p*-values accounting for multiple comparisons, with family-wise significance and confidence levels of 0.05).

To investigate correlations between scorings of the Mercuri grading and PDFF values, the Spearman correlation coefficient (*r_s_*) was computed. Additionally, to assess associations of quantitative assessments with BMRC scores of muscle strength of muscle groups responsible for hip flexion and extension as well as knee flexion and extension, *r_s_* was calculated between PDFF values and BMRC scores. Yet, to adjust correlation analyses for multiple comparisons, Bonferroni correction was applied to the *p*-values with a level of significance of *p* < 0.05/n (with n defined as the number of tested hypotheses).

## 3. Results

### 3.1. Study Cohort

Thirteen patients with NMD were included. Five patients suffered from DM2, five patients from LGMD2A, and three patients from adult Pompe disease (Table 2). Clinical examinations as well as MRI acquisitions with semi-quantitative and quantitative assessment were performed in all thirteen patients. There was a significant difference in muscle strength according to scores of the BMRC scale for knee flexion between patients with DM2, LGMD2A, and Pompe disease (Table 3).

### 3.2. Semi-Quantitative Mercuri Grading

Regarding the Mercuri scores for fatty infiltration, the scores ranged between 1.4 ± 0.5 (biceps femoris muscle) and 1.9 ± 1.2 (semitendinosus muscle) in DM2 patients, between 1.6 ± 0.5 (sartorius muscle) and 4.0 ± 0.0 (semimembranosus and semitendinosus muscles) in LGMD2A patients, and between 1.0 ± 0.0 (gracilis muscle) and 2.7 ± 0.6 (semimembranosus muscle) in patients with Pompe disease (Table 4).

### 3.3. PDFF of Thigh Muscles

**Figure 2:**
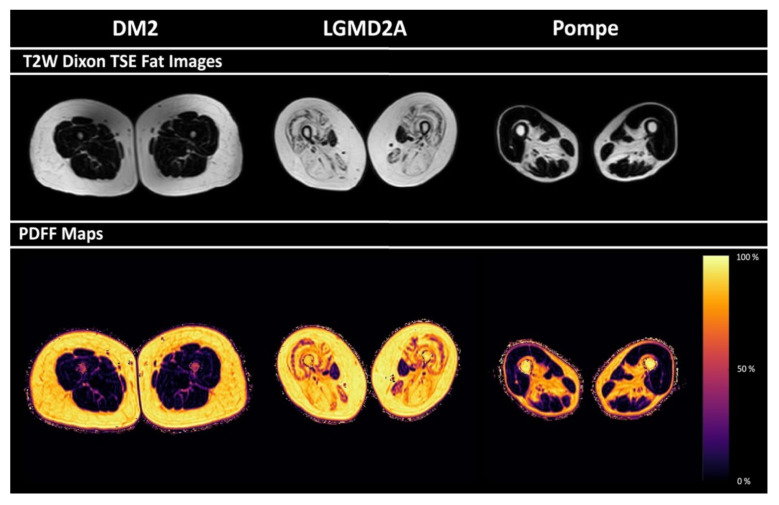
Representative T_2_-weighted DIXON turbo spin echo (TSE) fat images and proton density fat fraction (PDFF) maps for patients with myotonic dystrophy type 2 (DM2), limb girdle muscular dystrophy type 2A (LGMD2A), and Pompe disease. Disease-characteristic patterns of fatty infiltration are visible.

Representative PDFF maps for patients with DM2, LGMD2A, and Pompe disease are shown in Figure 2. Disease-characteristic patterns of fatty infiltration are visible. PDFF values obtained for different thigh muscles in the included patients ranged between 8.8 ± 1.6% (mean ± standard deviation) and 8.7% (median) (gracilis muscles in patients with Pompe disease) and 78.8 ± 3.8% (mean ± standard deviation) and 79.7%; 6.0% (median; interquartile range (IQR)) (semimembranosus muscles in patients with LGMD2A; Table 5). There were statistically significant differences in muscle PDFF between patients with DM2, LGMD2A, and Pompe disease for the gracilis (*p* = 0.04), biceps femoris (*p* < 0.01), semimembranosus (*p* < 0.01), and semitendinosus muscles (*p* < 0.01; Table 5).

### 3.4. Correlations between PDFF and Semi-Quantitative Mercuri Grading

Associations between PDFF and scores derived from Mercuri grading were statistically significant for almost all muscles of the thigh region (*r_s_* > 0.70, *p* < 0.05) except for the biceps femoris and vastus lateralis muscles (Table 6).

### 3.5. Correlations between PDFF and Muscle Strength

For PDFF, statistically significant correlations to muscle strength according to the BMRC scale were only found for muscle groups responsible for knee flexion (*r_s_* = −0.80, *p* < 0.01), whereas no statistically significant associations were revealed for hip flexion and extension or knee extension (Table 7).

## 4. Discussion

In the present study, quantitative imaging relying on chemical shift encoding-based water-fat MRI for PDFF determination was performed for the thigh muscles of patients suffering from three different types of NMD (DM2, LGMD2A, and Pompe disease). The PDFF of all muscles of the thigh region except for the biceps femoris and vastus lateralis muscles was significantly correlated to the Mercuri grading of fatty infiltration. Furthermore, muscle strength measured by the BMRC scale was significantly correlated to PDFF for muscles responsible for knee flexion.

In the diagnostic routine for patients with NMD, MRI using T_2_-weighted 2D DIXON TSE sequences allows a simultaneous qualitative assessment of fatty infiltration and edematous alterations in the patients’ muscle tissue [5,22,23,24,30,31,32]. Semi-quantitative grading based on MRI sequences using the Mercuri scale for fatty infiltration allows standardization and inter-scan, inter-reader, and inter-patient comparability [24,34], since the grading based on the semi-quantitative Mercuri scale shows substantial intra-reader agreement [32]. However, considerable effort has been invested to develop robust quantitative imaging techniques to overcome the remaining disadvantages of qualitative MRI with semi-quantitative grading, such as dependency of scores on individual readers and insensitivity to subtle changes in the muscle tissue by covering only a small dynamic range. Chemical shift encoding-based water-fat MRI allows the determination of PDFF as a marker for fatty infiltration [35,36,39]. The measurement of PDFF has been shown to be robust and precise. According to previous work, PDFF determination based on manual segmentation of single skeletal muscles is rather reproducible, showing an intra-reader reproducibility coefficient of below 2.2% and an inter-reader reproducibility coefficient below 0.5% for the PDFF measurements [33].

Semi-quantitative grading with the Mercuri scale, as used in the present study based on the T_2_-weighted 2D DIXON TSE fat images, showed disease-characteristic patterns of fatty changes in accordance with the literature for DM2, LGMD2A, and adult Pompe disease [10,11,12,13,14,15,16,17,18,19,20,21]. In particular, patients with DM2 showed relatively unaffected thigh muscles compared to patients with LGMD2A and Pompe disease, with the highest Mercuri scores for the semitendinosus muscle. In patients with LGMD2A, semitendinosus and semimembranosus muscles were highly fatty, with a relative sparing of the sartorius muscles. In patients with Pompe disease, the hamstring muscles (biceps femoris, semimembranosus, and semitendinosus muscles) were more affected than gracilis, rectus femoris, and sartorius muscles. Furthermore, PDFF values showed significant differences between LGMD2A, DM2, and Pompe disease. When concentrating on the biceps femoris, gracilis, semitendinosus, and semimembranosus muscles, the PDFF enabled a significant differentiation between LGMD2A, DM2, and Pompe disease. Particularly in post-hoc testing, PDFF of biceps femoris and semimembranosus muscles allowed a significant differentiation between DM2 and LGMD2A patients, and PDFF of semitendinosus muscles allowed a significant differentiation between patients diagnosed with LGMD2A and Pompe disease.

The correlations of the semi-quantitative grading with Mercuri scales with the obtained PDFF values revealed a strong correlation between Mercuri scores and PDFF values. When the PDFF is correlated to the Mercuri score of the same muscle summarized over all 13 patients, the values closely correlate for all muscles apart from biceps femoris and vastus lateralis muscles. The significant correlation between semi-quantitative and quantitative assessments of fat fraction suggests that PDFF could be a robust and reliable MRI-based biomarker for assessment of muscular fat content in NMD. Thereby, the slice thickness of the underlying chemical shift encoding-based water-fat sequence has a non-negligible influence on the cranio-caudal resolution. According to the work by Greve et al., a slice thickness of 8 mm enables the assessment of the variation between proximal, central, and distal muscle PDFF in patients with NMD [33]. When thicker slices are used, it has to be accounted for the influence of the slice thickness on the overall PDFF determination.

Additionally, measurements for PDFF correlated with muscle strength measurements for muscles responsible for knee flexion using the BMRC scale. However, the missing correlation of PDFF to knee extension and hip flexion/extension seems to contradict previous findings in a healthy cohort, where paraspinal muscle strength correlated with PDFF of the muscle tissue [37]. In this context, previous work stated that in diseased muscle tissue, the architectural and structural integrity of reorganization of the residual muscle fibers may have a bigger influence on the muscle function than mere fatty infiltration [43]. Also, a recent study found differences between muscle-strength measurements and MRI parameters, underscoring the difficulty to objectively measure muscle strength [40]. PDFF allows for detection of subtle changes of fatty infiltration [6,44]. These subtle changes detectable by quantitative MRI may not yet correlate with clinical functional measurements in early disease stages for all aspects of muscle function at the thigh region.

The present study has some limitations. First, the examined patient groups are small. However, the examined disease entities are rare [6,21,45]. Thus, low prevalence almost prohibits larger monocentric investigations with homogeneous MRI data acquisition using the same scanners and sequence protocols. Based on the presented preliminary data, in the future, large multi-center studies with harmonized MRI protocols are needed to further investigate the diagnostic performance of PDFF as an MRI-based biomarker. Second, the examined patients had different disease onset and disease duration, which have not been considered in the present study. However, disease characteristics should also be visible in disease groups with varying onsets, as especially fatty infiltration is known to be increasingly progressive in NMD. The pooling of patients with different disease onsets in this study is also owed to the already discussed low prevalence of NMD. Third, no additional functional test apart from examinations according to the BMRC scale was performed to assess muscle strength or function, such as a six-minute walking test. However, the BMRC scale is the most common assessment for muscle strength in the clinical routine and, therefore, the correlation between MRI parameters and BMRC scores may be of high interest.

## 5. Conclusions

In the small, investigated patient cohort, PDFF offers diagnostic precision similar to the clinically established Mercuri grading. Additionally, muscle strength was significantly correlated to PDFF for muscles subserving knee flexion. Based on these preliminary data, PDFF could be further considered as an MRI-based biomarker in the assessment of fatty infiltration of muscle tissue in NMD. Further studies with larger patient cohorts are needed to advance PDFF as an MRI-based biomarker in NMD, with advantages such as its greater dynamic range, enabling the assessment of subtler changes, the amplified objectivity, and the potential of direct correlation to muscle function for selected muscles.

## Figures and Tables

**Table 1:** Sequence parameters.

Scan Parameters	T_2_-Weighted 2D DIXON TSE	Six-Echo 3D Spoiled Gradient Echo
TR/TE/ΔTE (ms)	3725/100/1.0	10/1.17/0.9
FOV (mm^3^)	330 × 450 × 306	260 × 420 × 120
Acquisition voxel size (mm^3^)	2.5 × 2.7 × 6.0	3.2 × 2 × 4
Number of slices	26	30
Slice thickness (mm)	6.0	8.0
Slice gap (mm)	6.0	0.0
TSE factor	45	
Number of signal averages	2	1
Receiver bandwidth (Hz/pixel)		2325
Frequency direction		A/P
SENSE direction		L/R
SENSE reduction factor		2
Scan time per stack (s)	127	20

**Table 2:** Patient characteristics for patients with myotonic dystrophy type 2 (DM2), limb girdle muscular dystrophy type 2A (LGMD2A), and adult Pompe disease.

PATIENT ID	SEX	AGE	DISEASE	YEARS SINCE DIAGNOSIS	GENE MUTATION
**01**	m	52	DM2	1	*ZNF9/CNBP* gene: Tetranucleotide (CCTG) repeat
**02**	f	54	DM2	1	*ZNF9/CNBP* gene: Tetranucleotide (CCTG) repeat
**03**	f	61	DM2	4	*ZNF9/CNBP* gene: Tetranucleotide (CCTG) repeat
**04**	f	63	DM2	12	*ZNF9/CNBP* gene: Tetranucleotide (CCTG) repeat
**05**	f	66	DM2	11	*ZNF9/CNBP* gene: Tetranucleotide (CCTG) repeat
**06**	m	26	LGMD2A	2	c.1043delG c.1318C > T
**07**	f	45	LGMD2A	32	*CAPN3* gene: c.801 + 1G > A, c.1468C > G
**08**	f	47	LGMD2A	3	*CAPN3* gene: Exon 4: c598_612del Intron 13: c.1476-20C > G
**09**	f	49	LGMD2A	11	*CAPN3* gene: c.1099G > A, c.1322delG
**10**	f	52	LGMD2A	5	*CPN3* gene: c.759_761_del_GAA, c.1746-20C > G
**11**	m	48	Adult Pompe disease	7	*GAA* gene: c.-45T > G, c.1438-1G > C
**12**	f	76	Adult Pompe disease	26	*GAA* gene: c.-45T > G, c.1942G > A
**13**	m	84	Adult Pompe disease	8	*GAA* gene: c.-32-13T > G, c.1655T > C

**Table 3:** Age and muscle strength. Table providing details on age and muscle strength for muscle groups responsible for hip flexion and extension or knee flexion and extension (according to clinical examination considering the British Medical Research Council (BMRC) scale). Values are provided separately for patients with myotonic dystrophy type 2 (DM2), limb girdle muscular dystrophy type 2A (LGMD2A), and Pompe disease and are displayed as mean ± standard deviation. *P*-values for the comparison of the three groups as well as adjusted *p*-values (derived from post-hoc testing, *italics*) are shown (n.s.: not statistically significant, *p* > 0.05).

		DM2	LGMD2A	Pompe	*p*-Value (DM2 vs. LGMD2A vs. Pompe)	*Adjustedp*-Value (DM2 vs. LGMD2A)	*Adjustedp*-Value (DM2 vs. Pompe)	*Adjustedp*-Value (LGMD2A vs. Pompe)
**Age (in years)**		59.8 ± 6.1	44.3 ± 10.6	69.8 ± 19.2	0.02	*n.s.*	*n.s.*	*n.s.*
**Muscle strength (according to BMRC scale)**	**Hip flexion**	4.2 ± 0.5	3.2 ± 1.1	3.7 ± 0.6	n.s.	*n.s.*	*n.s.*	*n.s.*
	**Hip extension**	4.2 ± 0.5	3.4 ± 0.6	3.7 ± 0.6	n.s.	*n.s.*	*n.s.*	*n.s.*
	**Knee flexion**	4.6 ± 0.6	3.8 ± 0.5	5.0 ± 0.0	0.02	*n.s.*	*n.s.*	*0.03*
	**Knee extension**	4.8 ± 0.5	4.4 ± 0.9	4.7 ± 0.6	n.s.	*n.s.*	*n.s.*	*n.s.*

**Table 4:** Mercuri grading scores of fatty infiltration for muscles in the thigh region. Table showing Mercuri scores for the biceps femoris, gracilis, rectus femoris, sartorius, semimembranosus, semitendinosus, vastus intermedius, vastus lateralis, and vastus medialis muscles based on the T_2_-weighted two-dimensional (2D) DIXON turbo spin echo (TSE) fat images. Values are given as mean ± standard deviation. *P*-values for the comparison of the three groups as well as adjusted *p*-values (derived from post-hoc testing, *italics*) are shown (n.s.: not statistically significant, *p* > 0.05).

		DM2	LGMD2A	Pompe	*p*-Value (DM2 vs. LGMD2A vs. Pompe)	*Adjustedp*-Value (DM2 vs. LGMD2A)	*Adjustedp*-Value (DM2 vs. Pompe)	*Adjustedp*-Value (LGMD2A vs. Pompe)
**Mercuri grading score**	Biceps femoris	1.4 ± 0.5	2.2 ± 1.3	2.2 ± 1.3	n.s.	*n.s.*	*n.s.*	*n.s.*
	Gracilis	1.4 ± 0.9	2.0 ± 1.0	1.0 ± 0.0	n.s.	*n.s.*	*n.s.*	*n.s.*
	Rectus femoris	1.6 ± 0.9	2.1 ± 1.5	1.2 ± 0.3	n.s.	*n.s.*	*n.s.*	*n.s.*
	Sartorius	1.8 ± 1.3	1.6 ± 0.5	1.0 ± 0.0	n.s.	*n.s.*	*n.s.*	*n.s.*
	Semimembranosus	1.5 ± 0.5	4.0 ± 0.0	2.7 ± 0.6	<0.01	*<0.01*	*n.s.*	*n.s.*
	Semitendinosus	1.9 ± 1.2	4.0 ± 0.0	1.5 ± 0.5	0.02	*n.s.*	*n.s.*	*n.s.*
	Vastus intermedius	1.5 ± 1.1	2.8 ± 1.6	2.0 ± 1.0	n.s.	*n.s.*	*n.s.*	*n.s.*
	Vastus lateralis	1.7 ± 1.3	2.2 ± 1.3	1.8 ± 0.3	n.s.	*n.s.*	*n.s.*	*n.s.*
	Vastus medialis	1.8 ± 1.2	2.2 ± 1.3	2.5 ± 1.3	n.s.	*n.s.*	*n.s.*	*n.s.*

**Table 5:** Proton density fat fraction (PDFF) of muscles of the thigh region. Table showing PDFF (in %) for the biceps femoris, gracilis, rectus femoris, sartorius, semimembranosus, semitendinosus, vastus intermedius, vastus lateralis, and vastus medialis muscles. Values are given as mean ± standard deviation and median; interquartile range (IQR). *P*-values for the comparison of the three groups as well as adjusted *p*-values (derived from post-hoc testing, *italics*) are shown (n.s.: not statistically significant, *p* > 0.05).

		DM2	LGMD2A	Pompe	*p*-Value (DM2 vs. LGMD2A vs. Pompe)	*Adjustedp*-Value (DM2 vs. LGMD2A)	*Adjustedp*-Value (DM2 vs. Pompe)	*Adjustedp*-Value (LGMD2A vs. Pompe)
**PDFF** **(in %)**	Biceps femoris	15.5 ± 8.3 13.9; 12.2	67.6 ± 11.7 66.6; 22.8	18.7 ± 11.4 14.7; -	<0.01	*0.01*	*n.s.*	*n.s.*
	Gracilis	20.7 ± 14.2 15.2; 18,5	32.0 ± 21.1 35.6; 41.6	8.8 ± 1.6 8.7; -	0.04	*n.s.*	*n.s.*	*n.s.*
	Rectus femoris	17.7 ± 14.7 12.3; 20.5	28.5 ± 25.0 12.2; 45.0	12.6 ± 4.9 12.3; -	n.s.	*n.s.*	*n.s.*	*n.s.*
	Sartorius	24.4 ± 17.3 17.3; 21.8	19.4 ± 7.2 19.0; 10.92	11.2 ± 2.9 10.1; -	n.s.	*n.s.*	*n.s.*	*n.s.*
	Semimembranosus	16.9 ± 8.5 15.6; 13.4	78.8 ± 3.8 79.7; 6.0	32.1 ± 17.9 22.6; -	<0.01	*<0.01*	*n.s.*	*n.s.*
	Semitendinosus	18.6 ± 15.6 12.1; 22.9	76.6 ± 9.4 74.7; 17.9	9.1 ± 3.7 8.8; -	<0.01	*n.s.*	*n.s.*	*0.02*
	Vastus intermedius	17.2 ± 14.1 11.3; 18.2	30.1 ± 25.1 23.4; 46.2	23.0 ± 18.7 20.8; -	n.s.	*n.s.*	*n.s.*	*n.s.*
	Vastus lateralis	21.7 ± 18.6 14.0; 26.2	26.8 ± 27.8 9.3; 45.0	9.6 ± 2.7 8.2; -	n.s.	*n.s.*	*n.s.*	*n.s.*
	Vastus medialis	18.9 ± 17.9 10.5; 24.6	28.9 ± 24.4 25.8; 46.5	35.5 ± 32.8 31.1; -	n.s.	*n.s.*	*n.s.*	*n.s.*

**Table 6:** Correlation of the proton density fat fraction (PDFF) with semi-quantitative grading according to the Mercuri scale. Table showing the results of correlation analyses between PDFF values and Mercuri grading. Spearman correlation coefficients (*r*_s_) and corresponding *p*-values are shown separately for the biceps femoris, gracilis, rectus femoris, sartorius, semimembranosus, semitendinosus, vastus intermedius, vastus lateralis, and vastus medialis muscles. *: statistically significant after Bonferroni correction for multiple comparisons (*n* = 9).

	PDFF–Mercuri	
	*r_s_*	*p*-Value
**Biceps femoris**	0.38	0.19
**Gracilis**	0.81	<0.01*
**Rectus femoris**	0.87	<0.01*
**Sartorius**	0.71	0.01*
**Semimembranosus**	0.94	<0.01*
**Semitendinosus**	0.83	<0.01*
**Vastus intermedius**	0.86	<0.01*
**Vastus lateralis**	0.34	0.25
**Vastus medialis**	0.87	<0.01*

**Table 7:** Correlation of the proton density fat fraction (PDFF) with muscle strength. Table showing the results of correlation analyses between PDFF values and muscle strength as examined according to the British Medical Research Council (BMRC) scale. Spearman correlation coefficients (*r*_s_) and corresponding *p*-values are shown separately for muscle groups responsible for hip flexion and extension as well as knee flexion and extension. *: statistically significant after Bonferroni correction for multiple comparisons (*n* = 4).

	PDFF–BMRC	
	*r_s_*	*p*-Value
**Hip flexion**	−0.33	0.28
**Hip extension**	−0.36	0.23
**Knee flexion**	−0.80	<0.01*
**Knee extension**	−0.18	0.58

## Data Availability

The datasets generated during and/or analysed during the current study are available from the corresponding authors on reasonable request.

## Abbreviations

2D	Two-dimensional
3D	Three-dimensional
BMRC	British Medical Research Council
DM1	Myotonic dystrophy type 1
DM2	Myotonic dystrophy type 2
FOV	Field of view
FSHD	Facioscapulohumeral dystrophy
IQR	Interquartile range
LGMD2A	Limb girdle muscular dystrophy type 2A
MRI	Magnetic resonance imaging
NMD	Neuromuscular diseases
PACS	Picture Archiving and Communication System
PDFF	Proton density fat fraction
r_s_	Spearman correlation coefficient
ROI	Region of interest
SENSE	Sensitivity encoding
T_2w_	Water T_2_
TE	Echo time
TR	Repetition time
TSE	Turbo spin echo
